# Molecular underpinnings of prefrontal cortex development in rodents provide insights into the etiology of neurodevelopmental disorders

**DOI:** 10.1038/mp.2014.147

**Published:** 2014-12-02

**Authors:** D Schubert, G J M Martens, S M Kolk

**Affiliations:** 1 Department of Cognitive Neuroscience, Donders Institute for Brain, Cognition and Behaviour, Radboud University Nijmegen Medical Centre, Nijmegen, The Netherlands; 2 Department of Molecular Animal Physiology, Donders Institute for Brain, Cognition and Behaviour, Radboud University Nijmegen, Nijmegen, The Netherlands

## Abstract

The prefrontal cortex (PFC), seat of the highest-order cognitive functions, constitutes a conglomerate of highly specialized brain areas and has been implicated to have a role in the onset and installation of various neurodevelopmental disorders. The development of a properly functioning PFC is directed by transcription factors, guidance cues and other regulatory molecules and requires the intricate and temporal orchestration of a number of developmental processes. Disturbance or failure of any of these processes causing neurodevelopmental abnormalities within the PFC may contribute to several of the cognitive deficits seen in patients with neurodevelopmental disorders. In this review, we elaborate on the specific processes underlying prefrontal development, such as induction and patterning of the prefrontal area, proliferation, migration and axonal guidance of medial prefrontal progenitors, and their eventual efferent and afferent connections. We furthermore integrate for the first time the available knowledge from genome-wide studies that have revealed genes linked to neurodevelopmental disorders with experimental molecular evidence in rodents. The integrated data suggest that the pathogenic variants in the neurodevelopmental disorder-associated genes induce prefrontal cytoarchitectonical impairments. This enhances our understanding of the molecular mechanisms of prefrontal (mis)development underlying the four major neurodevelopmental disorders in humans, that is, intellectual disability, autism spectrum disorders, attention deficit hyperactivity disorder and schizophrenia, and may thus provide clues for the development of novel therapies.

## The prefrontal cortex in neurodevelopmental disorders

Neurodevelopmental disorders affect a large percentage of the population worldwide. Although the available drugs can alleviate some of the symptoms associated with these disorders, they are not curative and adverse drug reactions are often observed. In addition, many neurodevelopmental disorder-associated symptoms, especially cognitive symptoms, still cannot be treated effectively. To improve the prognosis of a given neurodevelopmental disorder, the effectiveness of existing therapies and the potential for finding new treatment strategies, detailed knowledge of the development and pathophysiology of the disorders is mandatory.^1, 2^ Neurodevelopmental disorders such as intellectual disability (ID), autism spectrum disorders (ASDs), attention deficit (hyperactivity) disorder (AD(H)D) and schizophrenia share particular cytoarchitectonical, connectional and functional features suggesting a similar neurodevelopmental origin. Unfortunately, for the most part, detailed molecular studies of developmental events within brain areas that are involved in the etiology of these neurodevelopmental disorders are still lacking.

A wealth of data indicates that the prefrontal cortex (PFC) contributes to the cognitive deficits or endophenotypes of many, if not all, neurodevelopmental disorders.^3, 4, 5, 6, 7, 8, 9, 10, 11, 12^ As a conglomerate of individually unique subareas, the PFC has a key role in the execution of higher-order cognitive functions, for example, language comprehension and cognitive functions involved in decision making such as planning and reasoning.^13, 14, 15, 16^ In this respect, the different subareas within the PFC mediate various processes including response inhibition, working memory, attention or autonomic control.^17, 18, 19, 20^ Furthermore, the medial regions of the PFC, the mPFC, such as the infralimbic, prelimbic and cingulated areas, have a role in the cognitive deficits of many neurodevelopmental disorders.^7, 11^

The main neurodevelopmental disorders—ID, ASDs, AD(H)D and schizophrenia—have a complex etiology involving a large number of genes and environmental factors that also affect prefrontal brain regions, including those of the mPFC. Although multiple genes have been found to be associated with each of these disorders, the actual function and involvement of individual genes in the developmental aspects of mPFC formation in particular are largely unknown. Abnormalities in the expression of these genes often lead to impaired or deviant functioning of several brain structures, including the mPFC, affecting behavior as previously shown in animal studies.^21, 22^

In the following, we will give an overview of the main neurodevelopmental disorders with a particular focus on the defects in the development of the mPFC, bearing in mind that areas other than the mPFC may also contribute to the etiology of the disorders.

### ID

The diagnostic category mental retardation groups a number of syndromes with severe ID that are associated with chromosomal abnormalities such as Down Syndrome (trisomy of chromosome 21), Prader–Willi and Angelman Syndromes, Williams–Beuren Syndrome, Smith–Magenis Syndrome, DiGeorge Syndrome and monosomy of chromosome 1p36.1.^23, 24, 25, 26^ Other ID syndromes show mild-to-moderate phenotypes and are associated with mutations, small insertions/deletions or copy number variations affecting a single gene, for example, fragile X syndrome, caused by a mutation in the *FMR1* gene^27, 28^ and Kleefstra syndrome, caused by a functional loss of the *EHMT1* gene.^29^ Most ID syndromes are associated with developmental deficits in general, including distorted development of the mPFC.^23, 24, 26, 30^ In this respect, during the development of the mPFC of ID patients, molecular/cellular defects have been shown to occur in (a) the **proliferation** of neuronal progenitor cells,^31, 32^ (b) **migration** of cortical neurons^33, 34, 35, 36, 37^ and (c) **synaptogenesis**.^32, 38, 39^

### ASDs

The ASDs include autism, Asperger's syndrome and ‘pervasive developmental disorder not otherwise specified' Diagnostic and Statistical Manual of Mental Disorders-5th edition (DSM-V). They constitute a group of wide-ranging neurodevelopmental disorders that are characterized by variable impairments in three core symptom domains, that is, reciprocal social interaction, (verbal and nonverbal) communication, and restricted, repetitive and stereotyped patterns of behavior, interests and activities.^40, 41, 42, 43^ Although many of these behavioral impairments are driven by deficits in basal ganglia and amygdala functioning, cognitive dysfunctions such as memory deficits and deficits in social interaction and perception are integrated by the mPFC.^44^ The neurodevelopmental basis underlying the defects in language and speech, which are often part of the diagnosis in ASDs relates to abnormalities in fronto-striatal functioning.^45, 46, 47, 48, 49^ Regarding the development of the mPFC of ASD patients, molecular/cellular defects have been reported to occur in (a) the **proliferation** of neuronal progenitor cells^50, 51^ resulting in macrocephalic and minicolumn pathology in several brain areas including the PFC,^3, 40, 42, 52, 53, 54^ (b) **migration** and differentiation of GABA ergic parvalbumin^+^ (PV^+^) interneurons toward the PFC,^36, 55, 56^ (c) **axon guidance**, as there seems to be a disconnection of long-distance axonal pathways^57, 58^ and (d) **synaptogenesis**, particularly of GABAergic synapses.^59, 60, 61^ Deficits in integration and early information processing can be explained by hyperconnectivity combined with slower synapses.^62^ Furthermore, there is evidence for amplified activation and density of microglia within the PFC of ASD patients.^57, 63, 64^

### AD(H)D

Inattention, hyperactivity/impulsivity and motivational/emotional dysregulation are the core symptom domains in AD(H)D. In AD(H)D patients, the mPFC-directed cognitive functions are affected and frequently of early onset.^65, 66, 67^ A delay in cortical maturation specifically in the most prefrontal areas and its connections to other brain areas has often been observed^68^ and there is increasing evidence that glutamate signaling is affected.^69^ During development, the PFC of patients with AD(H)D shows molecular/cellular defects in (a) the white matter, suggesting **axon guidance** deficits^70, 71, 72^ (b) dopaminergic and noradrenergic **connectivity** with the cerebellum and striatum^65, 67, 73, 74, 75, 76^ and (c) **synaptogenesis** influencing the electrophysiological properties and functioning of PFC neurons.^77, 78, 79^

### Schizophrenia

Schizophrenia is thought to affect mainly (social) cognition, but it usually is also associated with chronic problems of behavioral and emotional regulation.^80^ Schizophrenia is characterized by a breakdown of thought processes manifested as delusions and hallucinations (positive symptoms) and by poor emotional responsiveness, and disorganized thinking and speech (negative symptoms). People with schizophrenia are likely to have co-morbidities such as major depression and anxiety disorders. Furthermore, working and long-term memory, attention, executive functioning and speed of processing are often affected.^80^ All of these symptoms can at least to some extent be linked to (impaired) PFC functioning.^5, 12, 81, 82, 83, 84^ During development of the mPFC in schizophrenia patients, molecular/cellular defects may occur in the (a) **proliferation** of neuronal progenitor cells, as reflected by the observed severely decreased gray-matter volume,^85^ as well as of GABAergic PV^+^ interneurons,^86, 87^ (b) postnatal **pruning** of dendritic trees and **synapse** loss,^88, 89, 90, 91^ (c) general **connectivity** of various neurotransmitter systems such as the glutamate, GABA and dopamine systems together with a reduced connectivity with other cortical areas.^92, 93, 94, 95, 96, 97, 98, 99^

## Rodent models of neurodevelopmental disorders

Before one can start to develop better and more target-specific therapies for patients with neurodevelopmental disorders, it is necessary to first unravel elementary processes of brain development in adequate animal models and to understand subsequent developmental processes in those areas associated with the endophenotypes of neurodevelopmental disorders. In this way, fundamental hypotheses can be created and tested in relation to the etiology of these disorders. Such parallel approaches are crucial to eventually design optimal treatment strategies.

As mentioned before, although the PFC is often referred to as a single brain region, many subdivisions into distinct areas can be made, each of which possesses its own specific cytoarchitecture, cytochemistry, connectivity and functional properties. Defining these areas across species suffers from the fact that large interspecies differences exist in the layering per area, fueling the debate on whether or not rodents possess a region equivalent to the human PFC as they lack a granular zone in this area.^100, 101^ However, it should be noted that the formation of the general laminar pattern in the PFC shows a relation with phylogenesis: in ‘higher' mammalian species, such as primates and humans, PFC regions can be granular, that is, they possess a granular layer IV, as well as an agranular layer. The ‘lower' the species, the smaller the proportion of granular PFC regions (for reviews, see refs 100, 101). Thus the concept of homologous structures with similar functions may apply.

In this review, we will focus on the rodent mPFC and its structure–function relationships with connected brain areas in the context of neurodevelopmental disorders.^102, 103^ One example of a well-defined rodent model for neurodevelopmental disorders is the apomorphine-susceptible and apomorphine-unsusceptible Wistar rat. The behavioral impairments seen in the apomorphine-susceptible rats resemble features of schizophrenia.^104, 105, 106^ At least part of this phenotype can be attributed to the differences in the mesocorticolimbic projections.^107^

Furthermore, mouse models are ideally suited to study targeted molecular alterations.^102, 108, 109, 110, 111, 112, 113, 114^ In this way, genetic variants identified through association studies can be tested for their biological function and correlated with cognitive endophenotypes of human neurodevelopmental disorders. However, the traditional techniques of targeted mutation used in these kinds of model systems are systemic in nature and often result in inducing compensation mechanisms. Cre-Lox and knock-in systems still affect a large part of the brain, but can offer cell-type selective and temporally controlled strategies to achieve targeted mutations at different pre- and postnatal ages.^115^ Although *in utero* electroporation-mediated gene transfer spatially restrict gene repression or genetic rescues to early developmental time-points (app. E10-E17), virally mediated gene transfer can be performed pre- as well as postnatally.^116^ Furthermore, intersectional genetics (Flpe/Cre) to selectively mutate genes of interest in overlapping areas between a Cre and a Flpe allele (for example, Dlx5 Flpe and a region-specific Cre to selectively target GABAergic interneurons in a region of interest) increases the spatial selectivity of such approaches. Using these techniques, it is possible to knock down or rescue a particular gene in a specific part of the brain (for example, PFC) and at a specific time during brain development.

By employing various behavioral tasks, it is now possible to specifically test endophenotypes associated with mPFC function in rodent models, such as working memory, conditioned associative learning, attentional set shifting and reversal learning.^117, 118, 119, 120, 121, 122^ Consequently, by combining the targeted mutation with specific behavioral tests and instead of having to study a particular disease as a whole, one can now molecularly unravel the individual cognitive endophenotypes.^21, 22^ A further advantage of such an approach is that a causal inference can be made between the expression of a particular gene in a specific brain locus and one or more cognitive (endo)phenotypes, which is not yet possible in humans.

## Developmental aspects of PFC formation

**Figure 1:**
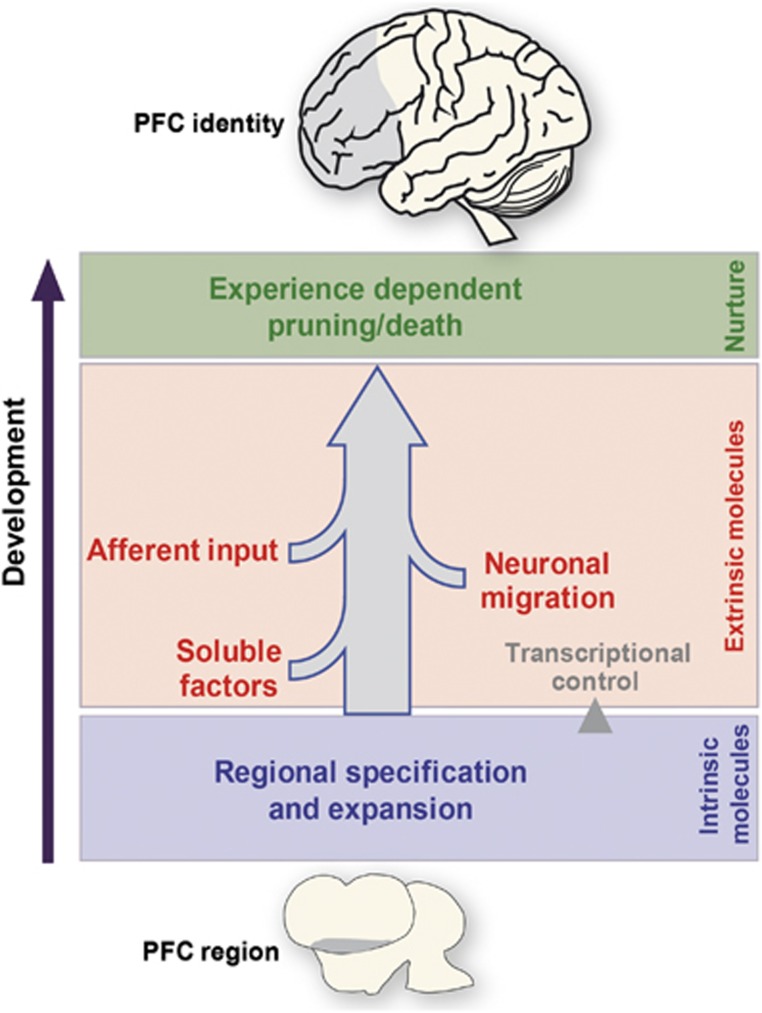
Bird's eye view of developmental events required for prefrontal cortex (PFC) formation. The identity of the PFC is sculpted over time by intrinsic developmental mechanisms such as expansion by proliferation and regional specification by the differential expression of intrinsic factors (e.g., transcription factors), indicated in blue. These intrinsic factors can control genes (transcriptional control) that affect other developmental events such as the expression and release of soluble morphogens, migration of neurons or guidance molecules that direct axons from other brain areas towards the PFC and vice versa to establish appropriate connectivity. These extrinsic factors are depicted in red. Pruning of appropriate connections and neuron death are under the control of external stimuli (green).

The PFC represents the functionally most advanced brain area with the longest period of maturation. This maturation includes proliferation and migration of neurons, growth of dendrites, the formation of neural micro- and macro-circuits through efferent/afferent axonal projections, and the fine-tuning of synaptic contacts and neuronal density steered by experience. This maturation process starts with an initial phase of cell division within an intrinsically specified PFC region, in which specific transcription factors (TFs) have a timing-critical role (Figure 1). Developmental events such as induction, migration and axon guidance are under the control of extrinsic cues and sculpt the identity of frontal areas. Appropriate cognitive behavior is fine-tuned over time by activity-dependent processes including sensory stimuli and social interactions, which in turn leads to pruning and cell death of unused connections.^123^ As a result, intricate convergence of connections with various other brain areas occurs, eventually creating the unique identity of the PFC and the subareas it encompasses (Figure 1). Here, the initial focus will be on the early developmental events of the (fore)brain as a whole and the molecules that are relevant during this phase. Although little is known about the early developmental characteristics of the PFC, many early principles and main mechanisms of forebrain compartmentalization and maturation are also applicable to PFC development. Important to keep in mind is the influence of external stimuli (for example, stress, drugs and hormones) that, if excessive, can lead to an altered development of the PFC and its connected areas.^123^ Thus, the knowledge about the genes that are involved in the structural and functional development of the (fore)brain and in particular the PFC is important for a better understanding of the molecular mechanisms underlying (disturbed) cognitive functions. Eventually, this knowledge may enable us to therapeutically intervene when this ‘developmental balance' is shifted toward neuropsychiatric disorder.

### Induction of (pre)frontal boundaries

The developmental progression of the forebrain starts with regional expansion through division of neuronal progenitor cells in proliferative zones lining the embryonic ventricles of the brain. The most anterior part of the neural tube develops into three primary vesicles even before the posterior section of the tube has formed: the prosencephalon (forebrain), mesencephalon (midbrain) and rhombencephalon (hindbrain).^124^ After closure, the neural tube is characterized by a sequence of swellings and constrictions along the anteroposterior axis, some of which subsequently develop into strict boundaries.^125^

**Figure 2:**
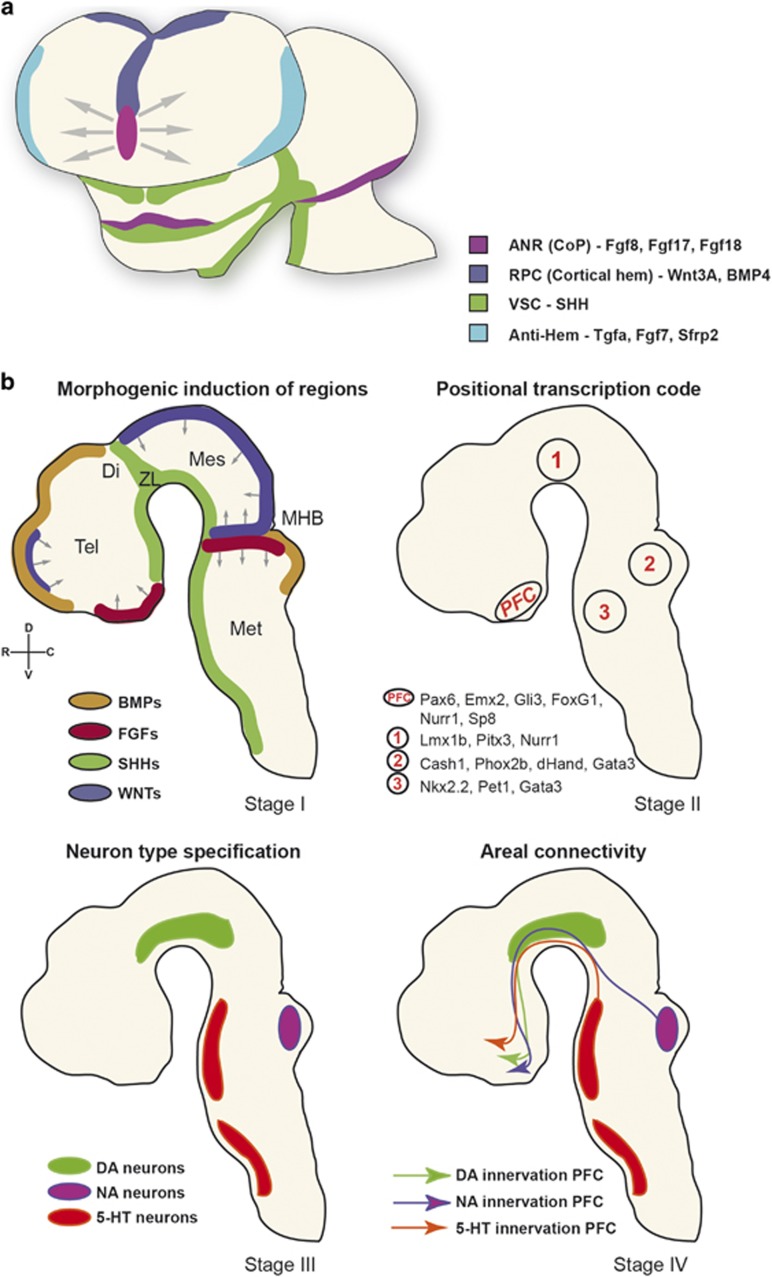
Molecular stages in the development of the PFC. (**a**) Schematic representation of the frontal view of a young (E11.5) mouse forebrain showing inductive influences (morphogens such as Fgfs, Wnts, SHH and BMPs; stage I). (**b**) Sagittal schematic views. These morphogens (stage I) have an effect on regional specification through intrinsic expression of transcription factors (stage II). This combinatorial code will have its effect on the cell-type specification of the major neurotransmitter systems (stage III). The neurotransmitter systems will connect to the PFC, shaping it and establishing the respective neural networks (stage IV). ANR, anterior neural ridge; DA, dopaminergic; DI, diencephalon; MES, mesencephalon; MET, metencephalon; MHB, mid-hindbrain border; NA, noradrenergic; PFC, prefrontal cortex; RPC, rostral patterning center; SHH, sonic hedgehog; Tel, telencephalon; VSC, ventral signaling center; ZL, zona limitans; 5-HT, serotonergic.

Except for the specific boundary compartment, the *zona limitans intrathalamica* (ZLI), no unique set of boundary markers has been identified for regions of the forebrain and most of the telencephalon develops in an unsegmented way.^125^ Anterior of the midbrain–hindbrain border (MHB) or isthmus, the diencephalon consists of three neuromeres (p1–p3) according to the so-called prosomeric model.^125, 126, 127^ The more anterior prosomeres (p4–p6) subdivide the secondary prosencephalon (hypothalamus and telencephalon).^128^ The boundaries that are created function to arrange and stabilize local signaling centers or ‘organizers' important for the early patterning of the embryonic brain (Figures 2a and b). Gradually, gradients of soluble morphogens and growth factors (Fgfs, BMPs, SHH and Wnts)^129, 130^ are secreted from signaling centers and regulate the graded expression of certain intrinsic TFs, a process that is called induction^131^ (Figures 2a and b).

Fgfs, especially Fgf8, Fgf17 and Fgf18 from the rostral patterning center (also called anterior neural ridge) provide, apart from their role in other areas, positional information on the presumptive prefrontal region along the rostro-caudal axis of the forebrain.^132, 133^ The dorsal patterning center or cortical hem secretes Bmp4/Wnt3A, which has a role in medial and dorsal pallium patterning,^134, 135, 136^ but in combination with SHH also steers prefrontal formation (Figures 2a and b). SHH is expressed by the ventral signaling center and regulates Fgf8 expression through the transcriptional repressor Gli3.^137, 138, 139, 140^ Absence of Fgf17 leads to a reduced PFC size and abnormal social behavior.^141, 142^ Thus, Bmp, Wnt and Fgf proteins all work coordinately to pattern the most rostral telencephalon.^139, 143^ Interference with each of the three Fgf receptor subtypes results in reduced numbers of either excitatory or inhibitory neurons, specifically in the prefrontal area and often resulting in altered behavior.^144, 145, 146, 147, 148, 149^

### Regional identity of the PFC through intrinsic patterning

The gradients of morphogens and signaling molecules from the early patterning centers impart positional information influencing the expression of intrinsic TFs (Figure 2b). These have a crucial role in the regionalization of the forebrain and correlate with morphologic boundaries, the so-called regional specification underlying the spatio-temporal control of postnatal arealization.^131, 150, 151, 152^ The regional identity that is created by the expression of TFs includes the final cell-type specification.^153^ The inductive signals provided by morphogens and signaling molecules regulate the combinatorial expression of TFs and other regulatory factors, resulting in the generation of specific neuronal subtypes^154, 155^ (Figure 2a and b).

The interaction between extrinsic growth factors and intrinsic TFs during the early developmental events evolves through rostral patterning by the factors Fgf8 and Fgf17 through the Fgf receptors. This Fgf-signaling promotes the expression of the TFs Foxg1, Six3, Sp8, Pax6, Erm (etv5), Er81 (etv1), Nkx2.1 and Pea3, and represses the expression of Coup-tf1 and Emx2 more caudally.^131, 133, 156^ Although it is most likely the expression of a combination of multiple TFs that underlies the identity of an area, there are a few individual TFs that are specifically linked to the development of the most rostral part of the cortex. The expression of the TFs Pax6 and Emx2, for example, is known to have a role in cortical identity in general.^131, 157, 158^ Yet, very few TFs are specifically expressed in and linked to early PFC development.

During the course of development, distinct neuronal cell types will express a variety of proteins that are involved in migration, targeting (for example, axon guidance) and specific neurotransmitter release. This set of proteins is unique for each cell type, thereby regulating the formation of functional areas.^159^ The expression of the respective genes (extrinsic genes) is under the control of a distinct combinatorial code of TFs generating neuronal diversity^160^(Figure 1 and Figure 2). Other TFs such as Rest4 and Nurr1 display increased expression in the PFC and are involved in various aspects of cognitive behavior.^161, 162^ Although an abundance of genome-wide expression data shows that specific TFs are expressed in later stages of PFC development, their downstream targets and functional relevance are largely unknown.^163, 164, 165, 166^ In fact, the existing data are now congruent with a model in which each neuronal cell type within the PFC (but also other areas) most likely uses an exclusive code of intrinsic genes to control the expression of extrinsic genes. This code is unique to each particular cell type essential for the sequential steps in development. The next level of complexity starts off when extrinsic mechanisms such as migration and afferent input begin to have a role in the development of the prefrontal areas.

### Proliferation and migration of PFC neurons

The PFC, like other cortical areas, expands by generating new neurons through (a)symmetric divisions of radial glia cells in the (sub)ventricular zone lining the ventricles.^167, 168^ During this process, reduction of the extrinsic morphogen Fgf8 results in less proliferation and more apoptosis, which ultimately changes the identity of the cortex.^132, 169, 170^ In particular Fgf has a determining role in the production of excitatory glutamatergic pyramidal neurons in the most anterior part of the cortex with deletion of the gene resulting in a reduced number of excitatory cortical neurons.^171^ Many TFs controlling the cell cycle, including cyclinD1, drive prefrontal expansion.^39^ Some newborn progenitors or intermediate progenitor cells expressing Tbr2 migrate to the subventricular zone to generate neurons. Lack of Tbr2 expression results in reduced cortical surface and thickness.^172, 173, 174, 175^ It is furthermore widely accepted that classical neurotransmitters such as dopamine and serotonin have an early role in controlling the neuron numbers within the PFC.^176, 177, 178^

The differential expression of TFs but also of adhesion and axon guidance molecules reflects a signage map for migrating neurons. The expression patterns are graded along the anterior–posterior and medial–lateral axes of the embryonic brain instructing neurons to establish functionally distinct lamina. During embryogenesis, most brain areas deploy radial migration in multiple waves as their major route to establish lamination within the structure.^167, 179, 180^ Radial glia cells, with their cell body within the ventricular zone, send out their glial processes toward the pial surface where they attach to the basal membrane. Newborn neurons that become (excitatory) projection neurons use the glial scaffold to migrate to their final place in the brain by using either somal translocation or locomotion.^167, 180, 181^ The ventricular zone generates the deeper layer neurons, including the subplate, layer VI and subsequently layer V projection neurons. Additionally, Cajal–Retzius neurons are generated within the cortical hem and to a lesser extent at other sites in the subpallium and septum. These layer I neurons express Reelin, a large secreted glycoprotein intricately involved in the inside-out laminar patterning of cortical neurons.^182, 183^ At later stages, the subventricular zone gives birth to neurons which migrate radially into the cortical plate past the deep layer neurons and form layers IV, III and II of the PFC, creating an inside-out pattern. Most of the projection neurons (80%) use glutamate as their neurotransmitter projecting to distant cortical and subcortical targets. The basic molecular developmental mechanisms that have been elucidated in rodent studies are in principle similar to those in humans, even though the human brain has gone through a series of additional evolutionary steps, including size, shape and gyrification modifications.^184, 185, 186^

### Migration of GABAergic interneurons towards the PFC

A small proportion of neurons, which includes the majority of GABAergic (GAD65/67^+^) interneurons originating from the ganglionic eminences, migrate tangentially to the cortical plate, then radially to reach their target lamina.^187^ The subpallial interneurons migrate via a lengthy route towards the PFC using directional cues to eventually position themselves between pyramidal projection neurons on which they synapse.^167, 188^ Medial ganglionic eminence-derived interneurons will generate PV and somatostatin interneurons that populate all cortical structures (as well as hippocampus, striatum, amygdala, etc). These interneurons are specified in the medial ganglionic eminence by the expression of Nkx2.1 and Lhx6 followed by Sox6 expression as they start migrating. In contrast, caudal ganglionic eminence-derived interneurons encompass all 5-HT3A-expressing interneurons of various morphology and physiology.^188^ The homeobox TFs Dlx1 and Dlx2 mainly regulate the maturation of GABAergic (inter)neurons within the ganglionic eminences, having the TF Arx as a downstream target.^133^ However, the combinatorial expression of TFs such as Olig2, Dlx5, Arx, Lhx6, Cux2, NPAS1 and MafB define the various subpopulations of interneurons within the subpallium that end up in the (prefrontal) cortex.^188, 189^ As development progresses, interneurons within the (prefrontal) cortex start to express transporters (GAT-1 and -3), VGAT and components of GABAergic synapses^190^ making them highly adaptive to the maturing PFC.

### Axon guidance, target selection and synapse formation of PFC neurons

The assembly of neuronal circuits during embryonic development relies upon the guidance of growing axons to their synaptic targets. To help them find their synaptic partners, developing axons are tipped with a highly motile sensory structure, the growth cone. Growth cones are instructed to follow predetermined trajectories by heterogeneously distributed guidance molecules in the extracellular environment. Binding of axon guidance molecules to receptor complexes on the growth cone surface initiates intracellular signaling events, which modulate growth cone morphology and directionality through local modifications of the cytoskeleton. Axon guidance molecules can act as attractants or repellents, that is, either directing growth cones toward a specific structure or preventing them from entering inappropriate regions. Furthermore, these cues exist as membrane-associated molecules acting at short ranges or as soluble agents with long-distance effects.^191, 192, 193, 194^ The responses of growing axons to particular cues, however, may change as they grow toward their final targets.^176^ For example, Semaphorin 3F is such a bidirectional guidance cue that, through binding with Neuropilin-2, initially repels dopaminergic axons from the rostral ventral tegmental area on their way to the mPFC, and later attracts and orients them within the mPFC.^176^ When the axonal growth cone has been guided to the proper target, synaptic contacts can be formed that are mediated by adhesion molecules such as the cadherins.^195, 196^ Newly formed synaptic contacts change their functional properties as development progresses and contribute to the maturation and functioning of an area.^197, 198^ Furthermore, the immature afferent projections are refined via the same guidance molecules in topography (pruning of branches), convergence (less efferent projections onto one cell) as well as postsynaptic compartment (less afferent dendritic innervation) in specific brain areas.^197, 198, 199^ Changes occurring in pyramidal morphology in terms of expansion of dendritic complexity are specifically apparent in layer III.^200^ Furthermore, during the first four postnatal weeks the local inhibitory interneuron networks in the mPFC undergo an extensive process of maturation, both at the level of intrinsic functional as well as network properties.^201, 202^ Given that inhibitory network activity is thought to contribute to the proper construction of cortical networks, the refinement of synaptic connectivity in inhibitory and excitatory networks leads to developmental plasticity and fine-tuning of complex behavior.

### Topographic map formation in PFC connectivity: parcellation versus lamination

**Figure 3:**
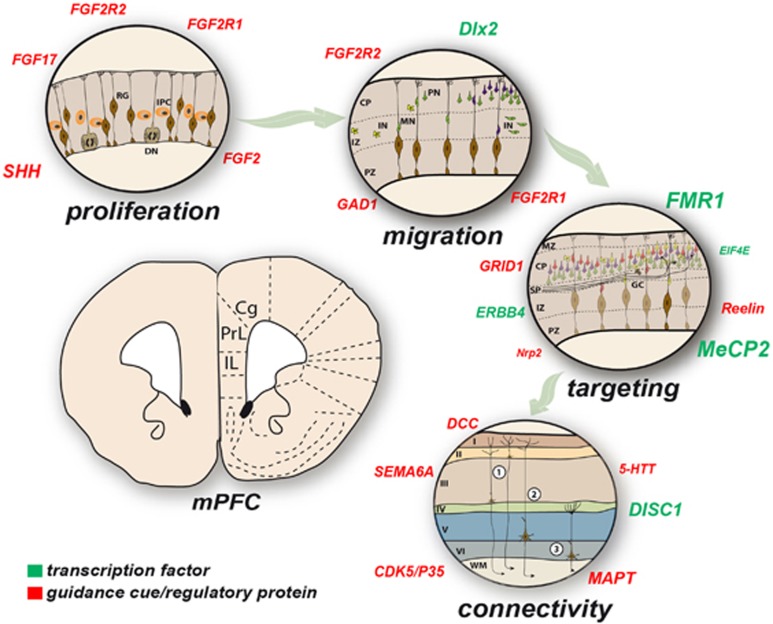
Neurodevelopmental disorder-associated genes that are involved in mPFC development. Various genes are associated with neurodevelopmental events in the mPFC (proliferation, migration, guidance targeting and connectivity) of which some can also be found in association studies with the four major neurodevelopmental disorders ID, ASDs, AD(H)D, schizophrenia. The letter size in the ‘cloud' of genes is indicative of the frequency of the gene associated with the various neurodevelopmental disorders connected to that particular neurodevelopmental event. Cg, cingulate cortex; CP, cortical plate; DN, dividing neuroblast; GC, growth cone; IL, infralimbic cortex; IN, interneuron; IPC, intermediate progenitor; IZ, intermediate zone; MN, migrating neuron, PN, post-mitotic neuron; PrL, prelimbic cortex; PZ, proliverative zone; RG, radial glia; (1) Commissural and corticocortical projection neurons, respectively; (2) subcerebral projection neurons to basal ganglia, diencephalon, midbrain, hindbrain and spinal cord; (3) corticothalamic projection neurons to mediodorsal thalamic targets; (2) and (3)=corticofugal.

As mentioned above, in rodents and other phylogenetically ‘higher' species, the PFC is not one homogeneous cortical region but is compartmentalized into a number of structurally and functionally distinct prefrontal areas, each of which is thought to possess characteristic input–output profiles. In general, the rodent PFC can be subdivided into medial, lateral and ventral sections. Within the medial portion, the anterior cingulate (Cg), prelimbic (PL) and infralimbic (IL) cortices (Figure 3) and dorsal peduncular cortex can be distinguished from dorsal to ventral.^203^ The lateral and ventral PFC consists of the orbitofrontal cortex and the agranular insular cortices.^204^ The different areas of the PFC are connected to various other brain regions through highly organized projections controlling decision-directed behavior.^205, 206, 207^

#### Input connectivity of the mPFC

In terms of the afferent connectivity of the mPFC, a comprehensive and detailed comparison of area-specific input connectivity is still lacking. The mPFC is known to receive long ascending projections from the ventral hippocampus,^208, 209^ from cholinergic neurons of the basal forebrain,^210, 211^ from dopaminergic neurons of the rostral part of the medial ventral tegmental area^176, 212, 213^ and from serotonergic/cholinergic neurons of the brainstem along a highly defined trajectory.^214, 215^ Functionally, the connection with the ventral hippocampus is thought to be of particular importance for the functioning of the mPFC during cognitive tasks.^216, 217^ The cholinergic and dopaminergic systems are considered to modulate mPFC activity and attentional performance.^218, 219^ Interestingly, the dopaminergic projections from the ventral tegmental area show strong laminar and cell-type specificity. They form dense contacts exclusively with interneurons in layers V and VI,^176, 213, 220, 221^ while for example projections from limbic and thalamic regions innervate both PV^+^ interneurons and pyramidal cells throughout layers II–VI.^222, 223, 224^ Furthermore, connections of the mPFC with both the basolateral amygdala^209, 225^ and the striatum are implicated in motivated behavior.^226, 227^ Interestingly, the long-range connections originating from the basolateral amygdala have been shown to not only be layer- but also cell-type specific. Neurons in the basolateral amygdala preferentially target layer II pyramidal neurons in the mPFC, such as PL, and amygdala, with which they can form reciprocal connections.^225, 228^

#### *Output connectivity of the mPFC*

As in other cortical areas, the long-range efferent connections of the PFC are mediated by excitatory projection neurons, that is, glutamatergic pyramidal cells. Depending on the PFC area, the pyramidal cells project to many structures such as the basal forebrain, olfactory and cortical structures, amygdala, striatum, (hypo)thalamus and the brainstem.^204, 215, 225, 226, 229^ In addition, prefrontal pyramidal neurons project to various subcortical areas thereby modulating dopaminergic, adrenergic, cholinergic and serotonergic projection systems.^101, 204^ The targets of the projection neurons show distinct layer specificity. Layer III pyramidal neurons connect the mPFC mainly to other cortical areas, whereas layers V and VI pyramidal cells project primarily to subcortical targets.^230, 231^ Furthermore, there is evidence for layer specificity of projections onto individual subcompartments of single brain structures. In terms of the nucleus accumbens, mPFC layer II pyramidal neurons preferentially innervate the core region, whereas neurons of deep layers V and VI innervate the core as well as the shell region.^232^

In contrast to the input connectivity, there is ample data demonstrating that the output connectivity properties of the mPFC are area dependent, which supports the notion that prefrontal areas are involved in modulating various aspects of cognitive behavior,^203, 204, 229^ not only in rodents but also in a number of other species.^220, 229, 230^ The dorsomedial areas of the PFC establish connections with the sensorimotor and association cortex, which are lacking in the ventral parts of the PFC. The ventral parts, however, establish relatively strong connections with the amygdaloid complex and limbic association cortices. Furthermore, the IL has been shown to mainly project to autonomic/visceral related sites, supporting its role in visceromotor activity,^204^ whereas the PL primarily innervates limbic sites that are thought to affect cognition.

## Future translational avenues of research

In summary, substantial progress has been made in the past decades toward understanding the etiology of neurodevelopmental disorders at the molecular, cellular and systems levels. Nevertheless, we have only just begun to thoroughly study the development of a conglomerate of specific brain areas that as a group define the PFC and that are involved in the etiology of these disorders. In this context, it is remarkable that the exact molecular orchestration of the development of the PFC is still largely unknown. What are the molecular mechanisms that create a correctly parcellated and layered PFC? How are the extensive and highly specific interactions between various signaling pathways that are connecting the individual areas fine-tuned and how can we manipulate these? We are also only beginning to shed light on the large variety of neuronal cells and their integration in prefrontal local and global networks, let alone that we would know all the molecules that guide their differentiation and projections.

To test targeted molecular variations, rodents have emerged as an excellent model. Animal models and functional assays are invaluable as it comes to decipher the exact functions of the large number of genes that are involved in the various aspects of PFC development, that is, induction of prefrontal boundaries, intrinsic patterning of the PFC, proliferation and migration of (pyramidal) PFC neurons, migration of GABAergic interneurons toward the PFC, axon guidance, target selection and synapse formation of PFC neurons, and PFC connectivity formation. Slowly, the view is emerging that some of these genes are identical to the susceptibility genes of neurodevelopmental disorders (Table 1). However, up to now only a few of the genes could be directly linked to one or more of the developmental events within the PFC as well as one or more of the four major neurodevelopmental disorders, that is, ID, ASDs, AD(H)D and/or schizophrenia.

Especially the availability of *in utero* electroporation-mediated gene transfer and other genetic approaches and hence the possibility to locally knock down or rescue particular genes will hopefully enable us to unravel the exact orchestration of brain areas such as those within the PFC in the near future. Such knowledge will assist in developing early intervention approaches by altering the susceptibility genes at a particular time and place, such that we deviate from the predetermined developmental path, even before the onset of the neurodevelopmental disorder(s) in question. Considering that individual susceptibility genes of neurodevelopmental disorders have often been found to be associated with multiple disorders, we can assume that several disorders share a common neurodevelopmental origin. It will be a challenge to dissect the individual genetic (and possibly even epigenetic) contributions to a disorder by using functional studies combined with behavioral tasks. For example, gene-environment interactions are crucial to distinguish between risk and vulnerability.

It is to be expected that in the coming years many more genes regulating developmental processes in the PFC and other brain structures will be linked to neurodevelopmental disorders and *vice versa*. Animal models, in which we can specifically alter gene expression in the PFC, can be instrumental for the understanding of the aetiopathological aspects of the disorder(s), as we can monitor the early disturbances that will eventually lead to defects in brain maturation and behavior. In order to move toward better and more preventive treatment of the neurodevelopmental disorders, bridges need to be built between disciplines such as combining genetic analyses of patients suffering from neurodevelopmental disorders with structural and functional brain imaging and in-depth molecular *in vitro* and *in vivo* approaches with cell and animal models. Exploring the molecular and cellular aspects during the progression of the disease process in animal models will clarify the pathological mechanisms, which in turn may provide clues to develop novel treatments for these disorders. The earlier during life and the more personalized the treatment strategies are applied, the better, alleviating symptoms at an early stage and reducing medical costs dramatically.

## Figures and Tables

**Table 1:** Commonalities in gene association between PFC developmental events and the four major neurodevelopmental disorders

*Gene*	*Involvement in PFC development*	*ID*	*ASDs*	*AD(H)D*	*Schizophrenia*
*Induction of prefrontal boundaries*					
*FGF17*	Fgf17 is secreted by the the rostral patterning center (RSC) and is involved in the induction of prefrontal boundaries.^141, 142, 233^		*Fgf17* knockout mice display deficits in specific social interactions that have been linked to ASDs.^142^		
*SHH*	Shh is secreted by the VSC and regulates the expression of Fgf8, which is involved in the induction of prefrontal boundaries.^137, 138, 139^	Mutations in *SHH* cause holoprosencephaly, a common forebrain malformation associated with craniofacial anomalies and MR.^234^	Significantly higher levels of serum SHH protein were found in children with autism.^235^	A mutation in *SHH* was found in two boys with ADHD.^236^	
					
*Proliferation and migration of PFC neurons*					
*FGF2*	Fgf2 has an important role in the production of glutamatergic pyramidal neurons in the (pre)frontal cortex.^237^			*Fgf2* knockout mice show hyperactivity.^238^	Serum FGF2 levels were found to be increased in people with schizophrenia.^239^
*FGFR1*	Fgfr1 is required for the proper number of glutamatergic pyramidal neurons in the frontal cortex.^144^	Dominant or recessive FGFR1 mutations are responsible for Hartsfield syndrome.^240^		Dysfunctional Fgfr1 signalling is associated with spontaneous hyperactivity.^144^	FGFR1 levels are higher in schizophrenia^241^ and th-fgfr1(tk-) transgenic mice exhibit behavior resembling human schizophrenia.^242^
*FGFR2*	Fgfr2 is involved in generating excitatory glutamatergic neurons in the mPFC.^147^	Mutations in *FGFR2* cause Crouzon's or Apert syndrome, which can be associated with MR.^243, 244^	Deletions of FGFR2 are associated with ASD.^245^	Some *Fgfr2* deficient mice display hyperactive behavior.^246^	A SNP flanking the FGFR2 gene is associated with schizophrenia.^247^
					
*Migration of GABAergic interneurons into the PFC*					
*DLX2*	Dlx2 controls interneurons migration toward frontal forebrain.^248^	Deletions of *DLX2* are associated with MR.^249^	*DLX2* shows genetic association with autism.^250^		
*GAD1*	Gad1 regulates the migration of GABA-ergic interneurons to the PFC.^251, 252^		Gad1 is an ASD susceptibility gene.^253, 254, 255, 256^		GAD1 expression is altered in schizophrenia patients and is considered a risk gene.^257, 258, 259^ Review: ref 260.
					
*Axon guidance, target selection and synapse formation of* *PFC neurons*					
*ERBB4*	Erbb4 regulates dendritic spine formation and density of PV+ interneurons in the PFC.^261, 262, 263, 264^	ERBB4 is associated with ID.^265^			Numerous studies implicate *ERBB4* as schizophrenia risk genes.^266, 267^ For reviews, see refs 268,269.
*EIF4E*	*Eif4e* has a role in synaptic function, dendritic spine density and synaptic plasticity of PFC neurons.^61^		*EIF4E* shows genetic association with autism.^270, 271, 272^ *Eif4e* transgenic mice display autism-like behaviors.^61, 273^		
*FMR1*	*Fmr1* functions in synaptogenesis of dendritic spines of PFC neurons.^62, 274, 275, 276, 277^	Mutations/deletions of *FMR1* cause Fragile X Syndrome, most common known hereditary cause of MR/ID and autism. Reviews: refs 28,30,278.	Mutations/deletions of *FMR1* cause Fragile X Syndrome, most common known hereditary cause of MR/ID and autism. Reviews: refs 279,280,281.	Human and animal models carrying the *FMR1* mutation display ADHD symptoms.^282, 283, 284, 285^	Reduced levels of FMR1 and mutations of associated genes in schizophrenia patients.^286, 287, 288^
*GRID1*	Grid1 has a role in synaptogenesis of PFC neurons.^289^		Genetic association^290^ and *Grid1* knockout mice show autism-like behavior.^289^		*GRID1* shows genetic association with schizophrenia and gray-matter reduction in patients.^291, 292^
*NRP2*	Nrp2 is involved in regulating axon guidance of PFC neurons.^293^		*NRP2* mutations are associated with autism.^294, 295^		
*RELN*	Reln is involved in regulating spine density and network formation.^296^	Disruption of *RELN* is associated with MR.^297^	*RELN* shows genetic association with autism. Reviews: refs 298,299,300.		*RELN* shows genetic association with schizophrenia. Reviews: refs 301,302,303.
*MECP2*	MeCP2 plays a critical role in the regulation of GABAergic transmission and cortical excitability of PFC pyramidal.^304^	MECP2 is associated with MR/ID and especially linked to Rett syndrome. Reviews: refs 305,306.	MECP2 is genetically linked to ASD.^307, 308^ Review: ref 309.		*De novo* mutations of MECP2 found in schizophrenia patients.^310, 311^
					
*PFC connectivity*					
*DCC*	DCC influences the prefrontal maturation and network formation with the dopaminergic midbrain.^312, 313^				Association between schizophrenia and genetic variation in DCC.^314^
*DISC1*	Disc1 KD is associated with dendritic abnormalities and affected cAMP signalling and hampers the mesocortical dopaminergic network formation.^21, 315^		DISC1 shows genetic association with autism.^316, 317, 318, 319^	DISC1 shows genetic association with ADHD in adults.^320^	DISC1 is a strong candidate gene for schizophrenia (recent reviews: refs 321,322,323.
					
*CDK5/P35*	*Cdk5r1* knockout mice display improper mesolimbic circuitry of the PFC.^324^			*Cdk5/P35* knockout mice display ADHD- like behavior.^324^	Lower levels of *CDK5/P35* in people with schizophrenia.^325, 326^
					
*MAPT*	Mutations in MAPT are associated with altered functional connectivity in the human PFC.^327^	MAPT CNVs and microdeletions in patients with MR.^328, 329, 330, 331^			
					
*SEMA6A*	Loss of *Sema6a* causes prefrontal loss of connectivity.^332^		*Sema6a* mutant mice display ASD-like behaviors.^332^		*Sema6a* mutant mice display schizophrenia-like behaviors.^332^
					
5-HTT	5-HTT is involved in proper raphe-prefrontal network formation.^215^				5-HTT is associated with schizophrenia.^333, 334^

Abbreviations: AD(H)D, attention deficit hyperactivity disorder; ASD, autism spectrum disorder; GABA, *γ*-aminobutyric acid; ID, intellectual disability; PFC, prefrontal cortex; PV^+^, parvalbumin^+^; RSC, rostral spinal cord; VSC, ventral signaling center.

Synopsis of the most cited genes that have been directly linked—through rodent studies—to one or more of the developmental events of PFC development (indicated in italics) and that have been directly genetically linked to the etiology of ID/MR, ASDs, AD(H)D and/or schizophrenia. Notes: (1) focus was on only those genes that were proven to be involved in prefrontal developmental events and not just expressed or involved in cortical development in general (e.g., Reelin); (2) A selection of references was made when more than three references were found.
